# Cholinergic Anti-Inflammatory Pathway and the Liver

**DOI:** 10.15171/apb.2017.063

**Published:** 2017-12-31

**Authors:** Khalil Hajiasgharzadeh, Behzad Baradaran

**Affiliations:** Immunology Research Center, Tabriz University of Medical Sciences, Tabriz, Iran.

**Keywords:** Hepatic vagus nerve, Autonomic dysfunction, Cirrhosis, Inflammatory reflex

## Abstract

The hepatic vagus branches innervate the liver and serve an important role in liver-brain connection. It appears that brain modulates inflammatory responses by activation of vagal efferent fibers. This activation and subsequent acetylcholine releases from vagus nerve terminals leads to inhibition of inflammatory cytokines through α7 nicotinic acetylcholine receptors (α7nAChRs) which located on the surface of different cell types such as liver Kupffer cells. This protective role of vagus-α7nAChR axis in liver diseases has been shown in several experimental studies. On the other hand, accumulated evidence clearly demonstrate that, autonomic dysfunction which is reduced functioning of both vagal and sympathetic nervous system, occurs during chronic liver disease and is well-known complication of patients suffering from cirrhosis. This review describes the impact and significance of cholinergic anti-inflammatory pathway in the liver and discusses about its disease-related dysfunction on the progression of cirrhosis. Considering the fact that sepsis is major cause of death in cirrhotic patients, convergence of these findings, may lead to designing novel therapeutic strategies in the field of chronic liver diseases management involving selective drug targeting and electrical nerve stimulation.

## Introduction

Chronic liver inflammation plays a pivotal role in physiopathology of liver cirrhosis.^1,2^ Many different anti-inflammatory strategies have been tried to reduce severity of cirrhosis or its complication. As a logical mechanism to reduce inflammation in the liver, some studies investigated the role of the anti-inflammatory neural pathway in modulation of hepatic inflammation. The function and dysfunction of such intrinsic protective mechanism during cirrhosis and some controversial findings about the involvement of the vagus nerve in hepatic disease are the subjects of current review.

### The inflammatory reflex

For the first time, Tracey and coworkers described a previously unrecognized role of efferent vagus nerve signaling in modulating inflammation,^3^ and the term “inflammatory reflex” was introduced in an influential report.^4^ The same group of investigators showed that such cholinergic inflammatory reflex requires the α7 nicotinic acetylcholine receptor (α7nAChR), a ligand-gated ion channel expressed on macrophages, lymphocytes, neurons and other cells.^5^ They reported that vagus nerve stimulation (VNS) inhibits tumor necrosis factor (TNF) synthesis in the wild-type mice, but fails to inhibit TNF synthesis in the α7-nicotinic receptor-knockout (α7KO) mice. Thus, they concluded that the α7nAChR subunit is essential for inhibiting cytokine synthesis by the cholinergic anti-inflammatory pathway (CAIP).^5^ Further investigations of this prototypical vagus nerve circuit, have shown that an acetylcholine-producing T cells are required for inhibition of cytokine production by VNS.^6^ Without these T-cells (e.g. in nude mice, who lack functional T-cells), vagal stimulation had no anti-inflammatory effect. Surprisingly in this nude mice, adoptive transfer of ACh producing T cells restores some vagal anti-inflammatory action.^6^ Considering these studies, in the latest model, the action potentials originating in the vagus nerve is propagated to the celiac ganglia, where the splenic nerve originates. Norepinephrine released from the splenic nerve interacts with β2-adrenergic receptors and causes the release of ACh from T cells containing functional choline acetyltransferase (T-ChAT cells). ACh interacts with α7nAChRs on macrophages and suppresses proinflammatory cytokine release and inflammation.^7^ Also, in another set of experiment the gastrointestinal CAIP was investigated and the results showed that the vagus nerve dampens intestinal inflammation by directly interacting with the intestine resident macrophages without the involvement of the spleen.^8^

### The inflammatory reflex in the liver

As the largest solid organ in the human body, the liver plays a central role in the regulation of homeostasis. The structure of the liver has profound implications for its immunological function. Liver’s immune cells play pivotal roles in the first defense line against invading intestinal pathogens and modulation of these inflammatory responses is critically important in this organ. Inappropriate responses, either too weak or too strong immune reactions may lead to spreading infection or tissue damage. Thus intrinsic immunomodulatory systems may be important in these settings.^9^

### The hepatic vagus branches

The liver is innervated by parasympathetic afferent and efferent fibers and emerging evidence indicates important role for this neural element in the regulation of hepatic functions. The vagus nerve as the longest cranial nerve is the primary link between liver and brain, and after leaving the brain, in the thorax, the left vagus branch forms the anterior vagal trunk enters abdomen through esophageal hiatus and the hepatic vagus branches originate from this anterior vagal trunk. These branches join the hepatic plexus and through it are distributed to the liver at its hilus and supply the organ.^10^ In some experimental models of chronic liver damage such as carbon tetrachloride (CCl_4_) induced cirrhotic rat livers, cholinergic nervous fibers are known to increase in the injured area.^11^

### Experimental evidence

To date, different animal models of systemic or local inflammation have been used to investigate the role of CAIP and α7nAChRs in modulation of inflammation in a variety of tissues including liver.^12^ In a recent study, Sakata et al. reported that in humans, the highest accumulation of α7nAChR in the body was observed in the liver.^13^ This finding may indicates the importance of vagus-α7nAChR axis in the liver. There is evidence showing the protective role of vagus-α7nAChR axis in liver diseases. Nishio et al. have indicated that vagus nerve via α7nAChRs on Kupffer cells regulates these cells activation in nonalcoholic steatohepatitis (NASH).^14^ In this study, wild-type (WT) mice undergoing hepatic vagotomy were fed a methionine- and choline-deficient (MCD) diet for 1 week and the results showed that hepatic vagotomy aggravated MCD diet-induced NASH, and also indicated that α7KO mice who were fed MCD diet for 1 week developed advanced NASH with highly activated Kupffer cells.^14^ Treatment of experimental NASH by nicotine which is a non-specific α7nAChR agonist was shown in another study.^15^ In addition to the experimental models of NASH development, the vagus nerve attenuates fulminant hepatitis induced by lipopolysaccharide (LPS) and D-galactosamine in mice.^16^ In another set of experiments, it was shown that hepatic vagus-α7nAChR axis attenuates hepatocyte damage upon ischemia–reperfusion (I/R) injury,^17,18^ and Fas-Induced Apoptosis.^19^ A study which carried out by Park and colleagues has indicated that cytoprotective mechanisms of nicotine in I/R injury exert via heme oxygenase-1 induction and intraperitoneally administration of nicotine can reduces the elevated levels of inflammatory cytokines after reperfusion.^18^ Fujing Li and colleagues indicated that PNU-282987, a selective α7nAChR agonist has protective effect in hepatic I/R injury by inhibition of high-mobility group box 1 (HMGB1) protein expression and nuclear factor kappa B (NF-kB) activation in mice.^20^ Activation of CAIP by long-term nicotine administration reduces sepsis-induced oxidative damage in several tissues including liver which appears to involve inhibition of neutrophil activity in the inflamed tissues.^21^ Likewise, high frequency VNS improves portal hypertension in cirrhotic rats.^22^ Clinical evidence indicates that when vagus nerve activity is deficient, inflammation is excessive.^23^ Vagotomy resulted in an enhanced influx of neutrophils and a marked increase in proinflammatory cytokine levels and liver damage.^24^ Hence, by considering these issues, the vagus-α7nAChR axis may play an important intrinsic protective role in centrally mediated hepatic immune responses. This protective role of inflammatory reflex should be noticed during liver transplantation surgery and CAIP agonists may be a potential target for sepsis after liver transplantation.

### Autonomic dysfunction (AD) in cirrhosis

**Figure 1:**
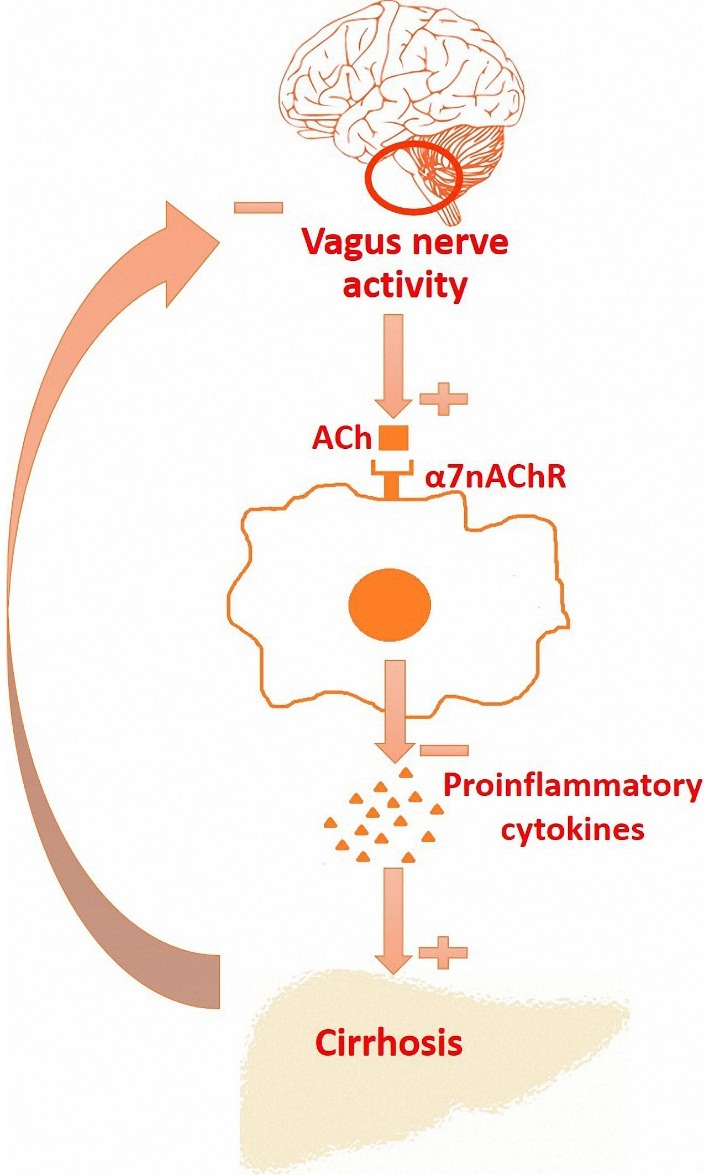
A hypothetical concept map about relationship between suppression of inflammatory responses through vagus-α7nAChR axis, worsening of cirrhosis due to inflammation and vagal hypoactivity in cirrhosis.

Autonomic dysfunction (AD) in the context of cirrhosis has been of increasing interest over the last 20 years. Accumulated evidence have shown that, one of the well-known complication and independent predictor of mortality of cirrhotic patients is AD.^25,26^ As the autonomic nervous system (ANS) activity involves multiple organs, autonomic dysfunction usually encompasses various and multiple disorders and may impair the quality of life. AD can be primary or secondary, acute or chronic and transient or progressive.^25^ There are many reports to indicate that liver cirrhosis is associated with AD and the vagal activity appears to be significantly lower in cirrhotic subjects in comparison with healthy individuals.^27,28^ Figure 1 shows an example of a concept map that describes the relationship between vagus-α7nAChR axis, worsening of cirrhosis due to inflammation and vagal hypoactivity in cirrhosis.

### Diagnosis and prevalence of AD in cirrhosis

AD can be assessed clinically by various autonomic function testing methods in patients with cirrhosis of different etiology. Some of these methods such as five standard cardiovascular autonomic reflex tests are the gold standard for identifying vagal or sympathetic function.^29^ By these tests parasympathetic integrity was explored by beat-to-beat variation during Deep Breathing (DB), Valsalva Maneuver (VM) and Lying-to-Standing (LS) tests. Sympathetic function was assessed by Orthostatic Hypotension (OH) and Sustained Handgrip (SH) tests.^30^ These tests provide information about the nature and severity of autonomic disorders and have been used in the clinical setting to generate a large body of evidence on AD.

The clinical picture of a patient with cirrhosis presenting with AD is similar to AD of any cause. Previous studies have been evaluated the prevalence of AD in patients with chronic liver disease. Cirrhosis, both alcoholic and nonalcoholic, has been reported to be associated with AD, as well as other hemodynamic and circulatory disturbances.^31,32^ Thuluvath and colleagues showed that 45% of patients with alcoholic liver disease and 43% with non-alcoholic liver disease had evidence of parasympathetic damage; 11% of patients with alcoholic liver disease and 12% with non-alcoholic liver disease had sympathetic damage.^31^ In other study, symptoms associated with cardiovascular AD were present in almost 70% of patients with primary biliary cirrhosis (PBC) and also in those in a precirrhotic stage.^33^ Hendriksen et al. found that vagal dysfunction is common in well compensated chronic liver disease and mortality rate in patients with vagal neuropathy was 30% compared with 6% in those with normal autonomic function.^34^ Gentile et al. reported AD in 60% of their patients.^32^ In this last study, the alterations of the parasympathetic function were significantly more frequent than those of the sympathetic function.^32^

In a study published in 2003, autonomic function using five standard tests were examined in 20 cirrhotics and 20 age and sex matched controls and was shown that sixteen (80%) of the cirrhotic subjects were found to have evidence of AD.^35^ Also in another study, AD observed in 67.7% of cirrhosis patients and in this study, parasympathetic dysfunction was more prevalent than sympathetic dysfunction (59.7% vs. 20.9%).^36^ The assessment of AD by standard cardiovascular autonomic reflexes is a little troublesome and have some drawbacks such as the need for active collaboration from the patients and short-term characteristic of measurements. Recently, Stevens and colleagues have investigated the presence of peripheral AD in PBC by using a novel and innovative microvascular optical technologies and provides evidence for the presence of peripheral AD in PBC patients.^37^

Along with AD assessment by standard tests, presence of AD in cirrhotic patients can be determined by linear (e.g. spectral analysis) and nonlinear (e.g. Poincare ´ plots) analysis of heart rate variability (HRV) and it has been shown that cirrhosis is accompanied by a significant decreases of HRV indexes.^27,28,38,39^ Spectral analysis of the R-R interval time series of heart beats can be carried out by fast Fourier transformation (FFT).^40^ In this method, a low-frequency component (LF), which reflects predominantly sympathetic activities, and high-frequency component (HF), which reflects the inhibition of vagal tone during inspiration can be assessed and LF/HF ratio is used as a measure of sympathovagal balance.^27,40^ Likewise, Poincare´ plots are used to distinguish the effects of vagal modulation from other causes of heart rate variation.^41^ Therefore, HRV analysis can be used as a measure of AD in cirrhotic patients. By 2-year follow-up periods of cirrhotic patients, Ates et al. showed that HRV measurements in cirrhotic patients were significantly much lower in nonsurvivors than in survivors.^42^ They conclude that increasing severity of cirrhosis is associated with a reduction in HRV.^42^ Other report showed that the relative risk of death increased by 7.7% in patients with cirrhosis for every 1-ms drop in a long-term HRV index as measured using Poincare’ plot.^27^

Despite many studies have used HRV analysis in diagnosis of AD in cirrhosis, on the other hand, some studies suggest that analysing HRV may not necessarily provide information on vagal activity when an end-organ hyporesponsiveness to cholinergic stimulation exists and this may pose limitations on the use of heart rate monitoring in this patient population.^43,44^

### Pathophysiology of AD in cirrhosis

Underlying mechanisms of AD in cirrhosis is largely unknown. Immunological and metabolic abnormalities may play a role, and some hypotheses are presented to explain the resultant decreased in autonomic function in cirrhosis. An interesting hypothesis is that AD is one of the consequences of the peripheral vasodilation associated with portal hypertension.^45^ Peripheral vasodilation stimulates the release of angiotensin and catecholamines and administration of an angiotensin converting enzyme inhibitor improves the HRV confirming that angiotensin II plays a role in AD.^46^ In other study, it is became clear that systemic inflammation exacerbates cytotoxic brain edema in bile duct ligated (BDL) rats and is a major component in the genesis of neural dysfunction in liver diseases.^47^ Moreover, the brain cholinergic system alterations have been examined in both cirrhotic patients and animal model of BDL induced liver damage and the results indicate an increased activity of the brain levels of acetylcholinesterase (AChE), while the activity of choline acetyltransferase (ChAT) remains unaffected.^48^ Recently, we showed that centrally cholinergic system activation prevents development of endotoxin tolerance in rat liver.^49^ These studies suggest that neural impairment may occur during liver disease due to peripheral and central causes; however greater understanding of the underlying mechanisms of AD of cirrhosis is required. Regardless of the etiology of disease, AD has been suggested to predict poor prognosis in patients with cirrhosis.^50^

### The inflammatory reflex and vagal dysfunction

AD is seen in patients with cirrhosis as well as diabetes mellitus, some autoimmune disorders and other chronic illnesses, such as HIV or Parkinson's disease. In a recent study, we investigated the role of vagus nerve in BDL-induced liver fibrosis in rats.^51^ We demonstrated by both immunohistochemistry and immunofluorescence staining that α7nAChR is mainly expressed in the hepatocytes of cirrhotic liver with minimum expression in control healthy liver. In this study, surgical or pharmacological inhibition of vagus nerve did not change the progression of hepatic fibrosis in BDL model of cirrhosis.^51^ This data may indicate that vagal neuropathy occurrence during liver damage which interrupts the protective role of vagus-α7nAChR axis in chronic liver disease. But it should be noted that, BDL model is characterized by higher damages to cholangiocytes and bile duct hyperplasia at peri-portal region, while recent studies which indicated the protective role of α7nAChR activation focused on ischemia/reperfusion injury and Fas-induced hepatitis in the liver which mainly involve hepatocytes.^17-19^ Therefore, this hepatocytes injury models may be more appropriate for investigating of vagus-α7nAChR axis in the liver. Interestingly, in a recent study, Gergalova et al. showed that α7nAChR is expressed in hepatic mitochondrial outer membrane and regulates early proapoptotic events like cytochrome c release and has protective anti-apoptotic effects.^52^ Thus, in addition to plasma membrane α7nAChRs, there exist mitochondrial α7nAChRs which control mitochondria functions and their apoptotic susceptibility.^52^

### Points of dispute

Conversely to inflammatory reflex hypothesis, some other studies indicate the cytotoxic and fibrogenic effects of nicotine by means of nicotinic acetylcholine receptor that aggravates the process of liver disease.^53,54^ It has been shown that nicotine induces fibrogenic changes in human liver via nicotinic acetylcholine receptors.^55^ Recently Zhou et al. indicated liver detrimental effect of nicotine by both in vivo (thioacetamide (TAA)-induced liver damage) and in vitro (with HepG2 and LX-2 cell lines) experiments in mice.^53^ In this study, oral administration of nicotine significantly aggravated TAA-induced hepatic damage through enhancing TGF-β secretion and oxidative stress.^53^ Soeda et al. have shown that hepatic stellate cells express α7nAChRs and nicotine at levels in smokers’ blood is pro-fibrogenic, through actions on these expressed receptors.^55^ Consistent with these findings, we can find some protective effects of selective hepatic vagotomy against BDL-induced liver damage. In our study, vagotomy could induce a significant decrease in elevated serum AST in vagotomized rats compare to non-vagotomized BDL rats.^51^

In addition, vagus nerve releases ACh and subsequent ACh induces fibrogenic effects via muscarinic acetylcholine receptors in NASH and in primary human hepatic stellate cells (hHSC).^56^ Morgan and colleagues showed that cultured hHSC produce ChAT and AChE, as well as secreting ACh and suggested that ACh is an autocrine growth factor for HSC proliferation and fibrogenesis.^56^ In another study, in the BDL model, vagotomy induces the disappearance of muscarinic-type-3 (M3) ACh receptor, a marked impairment of cholangiocyte proliferation and activation of apoptotic cell death.^57^ When BDL rats were treated with forskolin, an activator of adenyl cyclase, cAMP intracellular levels were maintained and vagotomy failed to impair cholangiocyte proliferation and did not induce apoptosis.^57^ In another hand, Kiba et al. demonstrated that vagal hyperactivity after ventromedial hypothalamic lesioning stimulates Fas mediated apoptosis through the cholinergic system in the rat liver, which is in contradiction with inflammatory reflex hypothesis.^58^

### Conclusion and perspective

The liver is innervated by parasympathetic nerve fibers and both muscarinic and nicotinic acetylcholine receptors are expressed in the liver and exert many different functions. Chronic inflammation plays a pivotal role in many disease states including liver cirrhosis and cancer.^59,60^ In other hand sepsis is the major cause of death in patients suffering from cirrhosis.^61^ This reciprocal interaction between inflammation and cirrhosis persuade physicians to applying novel therapeutic strategies, for example strategies based on RNA interference technology in diseases management.^62,63^ Also, considering the increased frequency of liver transplantation, understanding the role of parasympathetic nerve fibers in liver is of particular importance since the human liver is not re-innervate after transplantation. It appears that brain modulates inflammatory responses by activation of vagal efferent fibers. Therefore, the parasympathetic system may modulate the inflammatory response in real time. However, during the pathologic conditions such as liver cirrhosis, the brain-liver connection through vagus nerve may not play its role in modulation of immune responses.

This may help us to explain why AD and specially hypoactivity of the vagal nerve contributes to poor survival in cirrhosis. Since, tight regulation of inflammatory responses is vital to ensure that it does not spin out of control and becomes harmful to the host; disruption of inflammatory reflex by vagal neuropathy may be the cause of significant mortality rate of cirrhotic patients due to sepsis. In this sense, vagus nerve stimulation appeared as a possible efficient procedure to minimize inflammation. The relationship between vagus nerve activity and inflammation in the process of cirrhosis is complex and studying the interaction between them will help developing novel therapeutic strategies for cirrhotic patients.

## Acknowledgments

The authors would like to thank Dr. Ali R. Mani (University College London) for carefully reading and insightful comments on the paper.

## Ethical Issues

Not applicable.

## Conflict of Interest

Authors declare no conflict of interest in this study.
